# Editorial: Family and school-based interventions to increase adolescents' leisure-time physical activity

**DOI:** 10.3389/fpsyg.2023.1208687

**Published:** 2023-06-13

**Authors:** Henri Tilga, Andre Koka, Javier Sevil-Serrano

**Affiliations:** ^1^Institute of Sport Sciences and Physiotherapy, Faculty of Medicine, University of Tartu, Tartu, Estonia; ^2^Faculty of Teacher Training, University of Extremadura, Cáceres, Spain

**Keywords:** physical activity, parental supervision, autonomy support, physical education, intervention

## Introduction

The role of physical activity, extracurricular activities, and parental involvement in children's academic success and emotional wellbeing has become increasingly crucial in recent years (Harnois-Leblanc et al., 2023). The four studies discussed in this editorial provide valuable insight into the importance of parental support, autonomy, and physical activity in fostering children's cognitive and non-cognitive skill development. These findings have significant implications for teachers, policymakers, and parents seeking to promote holistic child development.

## Physical activity: jump rope-based homework intervention

The first study by Huang et al. highlights the potential of physical activity homework in promoting after-school fitness among middle school students. The study employed a randomized controlled trial involving 93 middle school students assigned to either a homework group (HG) or a control group (CG). The HG participants completed jump rope homework three times per week for 12 weeks, while the CG attended weekly health education classes. The results suggested a significant improvement in speed, endurance, power, and core muscular endurance among students in the HG, emphasizing the importance of incorporating PA homework to enhance students' physical fitness.

## Parental supervision and support in visual art activities

The second study by Tadesse et al. focuses on the role of parental support and guidance in managing children's out-of-school visual art activities and academic work time in a Chinese context. The research involved over 2,400 primary school students in one Chinese province, examining the impact of parental supervision on the benefits of extracurricular activities for children's cognitive and non-cognitive wellbeing. The findings challenge the traditional stereotype of aggressive and overbearing Chinese parents, revealing that contemporary Chinese parents value their children's interests. However, the study found no substantial value in children's participation in out-of-school visual art activities for promoting academic performance, even with genuine interest and supportive parenting.

## The impact of visual art activities on academic attainment

The third study by Deer et al. (2023) further investigates the effects of out-of-school visual art activities on children's academic attainment across economically (dis)advantaged children in southwestern China. The large-scale survey involving 1,624 participants revealed that contemporary Chinese parents, regardless of social class, were dedicated to providing their children with out-of-school activities. Nevertheless, the study emphasizes the need for lower-class parents to understand that spending time with their children during these activities is more beneficial than simply purchasing access to them.

## Autonomy-supportive teaching and affective-emotional perception in physical education

The fourth study by Leisterer and Paschold explores the impact of autonomy-supportive teaching in physical education (PE) on students' affective-emotional perception. This quasi-experiment involved 57 German students who were exposed to high autonomy-supportive (PE_high), low autonomy-supportive (PE_low), or controlling (PE_control) teaching styles in PE classes. The results demonstrated that students who participated in PE_high experienced significantly more positive affective valence and enjoyment than those in the PE_low and PE_control groups. Consequently, the study recommends PE teachers adopt a high autonomy-supportive teaching style, incorporating free choices, social interaction, and informative feedback, to improve students' positive affective-emotional perception and engagement in physical activity.

## Conclusions

Collectively, these four studies highlight the importance of a school community agents to nurturing children's academic and emotional development. Physical activity, as exemplified by the jump rope-based homework intervention, plays a vital role in enhancing students' fitness and overall wellbeing. Moreover, parental support and involvement in children's extracurricular activities, such as visual art, can promote cognitive and non-cognitive skill development. The findings also emphasize the significance of autonomy-supportive teaching in fostering positive emotional perceptions and enjoyment in PE classes. These studies serve as a reminder to parents, teachers, and policymakers of the need to create a supportive and well-rounded educational environment for children growth. By fostering a holistic approach that embraces physical activity, extracurricular activities, parental involvement, and/or autonomy-supportive teaching, children can thrive both academically and emotionally.

It is essential for teachers to incorporate physical activity into the school curriculum and encourage students to engage in regular exercise, as demonstrated by the jump rope-based homework intervention. Such activities not only contribute to students' physical health but can also positively influence their cognitive functioning and emotional wellbeing.

Parental involvement, as shown in the second and third studies, is a critical factor in children's academic success and personal growth. Parents should strive to understand and support their children's interests and ensure a balanced approach to manage their time between academics and extracurricular activities. Schools and communities should provide accessible opportunities for economically disadvantaged children to engage in enriching activities, emphasizing the importance of parental presence and engagement during these activities.

The fourth study underscores the value of autonomy-supportive teaching in fostering a positive learning environment for students. By allowing students to have a sense of ownership and control over their learning experience, teachers can enhance students' motivation, enjoyment, and emotional wellbeing in PE classes (Vasconcellos et al., 2020; Ahmadi et al., 2023). This teaching approach can extend to other subjects as well, promoting a supportive and engaging learning environment across the entire school curriculum.

In conclusion, the studies discussed in this editorial shed light on the multifaceted nature of child development and the critical role of different school community agents on physical, psychological, and academic indicators. By adopting these research-based strategies, parents, teachers, and policymakers can work together to create a nurturing and well-rounded educational environment that fosters the holistic development of children, preparing them for a successful and fulfilling future.

## Author contributions

All authors listed have made a substantial, direct, and intellectual contribution to the work and approved it for publication.
